# Osteoporosis-Pseudoglioma in a Mauritanian Child due to a Novel Mutation in* LRP5*

**DOI:** 10.1155/2016/9814928

**Published:** 2016-01-19

**Authors:** Noura Biha, S. M. Ghaber, M. M. Hacen, Corinne Collet

**Affiliations:** ^1^Rheumatology Department, Nouakchott Military Hospital, Mauritania; ^2^Faculté de Médecine de Nouakchott, Mauritania; ^3^Service des Laboratoires, Centre Hospitalier National de Nouakchott, Mauritania; ^4^Service de Chirurgie Orthopédique, Hôpital Militaire de Nouakchott, Mauritania; ^5^Assistance Publique-Hôpitaux de Paris, Hôpital Lariboisière, Laboratoire de Biochimie et de Biologie Moléculaire, Paris, France

## Abstract

Osteoporosis-pseudoglioma (OPPG) syndrome is a very rare autosomal recessive disorder, caused by mutations in the low-density lipoprotein receptor-related protein 5 (LRP5) gene. It manifests by severe juvenile osteoporosis with congenital or infancy-onset visual loss. We describe a case of OPPG due to novel mutation in LRP5 gene, occurring in a female Mauritanian child. This 10-year-old female child was born blind, and after then multiple fragility fractures appeared. PCR amplification and sequencing revealed a novel homozygous nonsense mutation in exon 10 of the LRP5 gene (c.2270G>A; pTrP757^⁎^); this mutation leads to the production of a truncated protein containing 757 amino acids instead of 1615, located in the third *β*-propeller domain of the LRP5 protein. Both parents were heterozygous for the mutation. This is the first case of the OPPG described in black Africans, which broadens the spectrum of LRP5 gene mutations in OPPG.

## 1. Introduction

Osteoporosis-pseudoglioma (OPPG) syndrome (OPPG, OMIM 259770) is a very rare autosomal recessive disorder. It combines severe juvenile osteoporosis with congenital blindness. This serious disease is caused by loss-of-function mutations in the low-density lipoprotein receptor-related protein 5 (LRP5) gene [1]. The LRP5 is a coreceptor of* Wnt*, situated on the osteoblast cell; it located between two other receptors named Frizzled (Fz) and Kremen family that plays a central role in* Wnt/-catenin* canonical pathway [2].

OPPG was first described in 1931 [3]. Gong et al. [4] had first identified loss of function mutation of the LRP5 gene leading to osteoporosis pseudoglioma (OPPG). To date, thirty mutations have been described in OPPG including fourteen homozygous mutations, principally located in the second and third beta-propeller domains of LRP5 [1, 2], which have a high affinity with* wnt* ligand [5].

Besides, gain-function mutations of LRP5 lead to high bone mass (HBM) (Familial High Bone Mass Syndrome) [6], osteopetrosis autosomal dominant type 1 [7], and osteosclerosis [8]. low-density lipoprotein receptor-related protein* 5* (*LRP5*), located on chromosome 11q13, has 23 coding exons. LRP5 cDNA which contains 4845 base pairs encodes a 1615-amino acid protein [9]. To date, only sixty OPPG cases were identified [2]. Here we describe a case of OPPG due to a novel LRP5 mutation occurring in a female Mauritanian child.

## 2. Case

**Figure 1:**
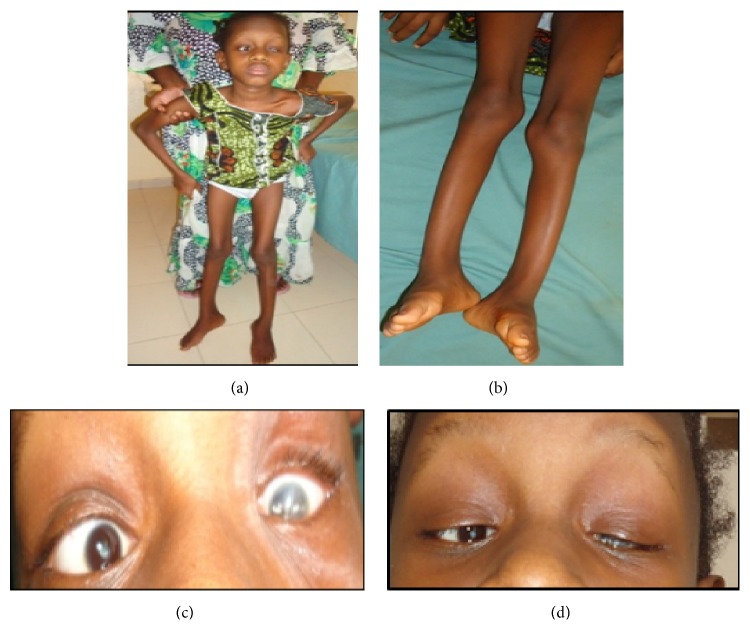
Photograph of child with OPPG showing corneal opacity, microphthalmia (c, d), and incurvation of Tibia (a, b).

**Figure 2:**
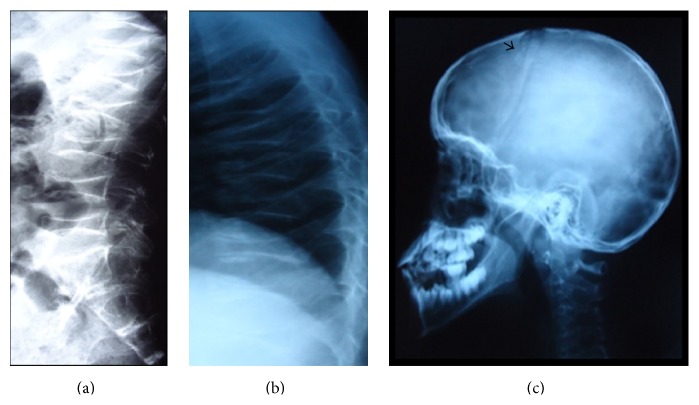
Lateral spine radiographs showing severe osteopenia, platyspondyly (a, b), Skull X-ray revealed wormian bone ↘ (c).

This report concerns a ten-year-old Mauritanian female child, who was referred by orthopedics service for assessment of fragility fractures. She was born to consanguineous parents. Congenital blindness was diagnosed at birth. She then presented with five broken limbs (humerus, wrist, ankle, and femur) after a fall from standing height, which premiered at the age of 5 years. Since femur fracture, she did not walk again. On clinical examination, we observed microphthalmia, corneal opacity (Figure 1), dorsal kyphosis, and incurvation of tibias and lower limb length inequality (Summarized Figure 1). Her weight was 15 kg and size was 117 cm, both far below the second percentile for her age. Neurological examination was normal. Serum calcium, phosphate, alkaline phosphatase, creatinine, and 25 OH vitamin D3 were all normal. Radiographs showed diffuse bone demineralization, multiple vertebral fractures, and platyspondyly (Figure 2). Bone mineral density (BMD) revealed a *Z* score of −5.5 at the spine.

## 3. Sequencing Analysis

**Figure 3:**
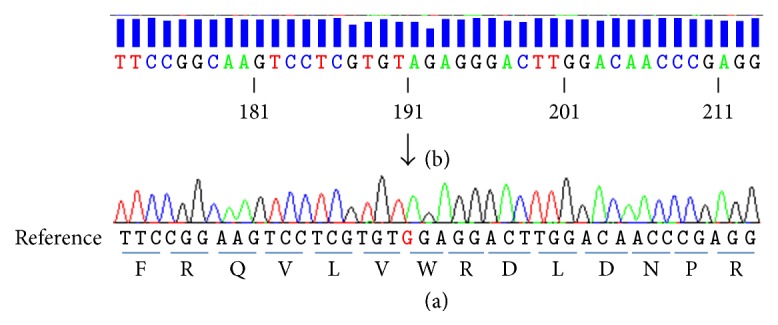
*c.2270G>A, p.Trp757* mutation in the LRP5 gene. (a) cDNA reference sequence for LRP5. (b) cDNA of the proband revealed a G-* *-* *-* *-A substitution at nucleotide 2270, resulting in a trp757-to-stop codon.

Written informed consent was obtained from her parents. Genomic DNA was extracted from the patient peripheral blood leukocytes using QIAamp DNA blood midi kit (QIAGEN). We screened all the 23 coding exons of* LRP5* for the case and for her parents. The PCR products were sequenced on both strands with ABI Prism 3130 Genetic Analyzer (Life Technologies, Saint-Aubin, France). Sequences were analyzed using SeqScape 4.0 software (Life Technologies) and compared with the genomic reference sequence (NG_015835.1) for* LRP5*. Mutation nomenclature was based on HGVS nomenclature guidelines [http://www.hgvs.org/mutnomen/] and exonic numbering was based on genomic reference (Figure 3).

## 4. Results

PCR amplification and sequencing revealed a novel nonsense mutation in exon 10 of the LRP5 gene (c.2270G>A; pTrP757^*∗*^). It produces a truncated protein containing 757 amino acids instead of 1615. This mutation is located in the third beta-propeller domain (YWTD repeat) in the extracellular domain of the receptor. Both parents were heterozygous for the mutation.

## 5. Discussion

OPPG syndrome is extremely rare genetic disorders, transmitted by autosomal recessive, associating congenital or infancy-onset visual loss with early-onset severe osteoporosis [6]. Other clinical manifestations can be observed like a muscular hypotonia, ligamentous laxity, mental retardation, and obesity [10]. Ocular abnormalities is due to persistence of the fetal ocular fibrovascular system, which seems due to a failure of macrophage-induced endothelial cell apoptosis (which needs* wnt* protein) [11].

We described here the case of a 10-year-old Mauritanian female child, who had a clinical OPPG phenotype. Molecular analysis identified a novel homozygous nonsense mutation in the LRP5 gene, leading to the substitution of G-to-A at nucleotide 2270 in exon 10, resulting in a trp757-to-stop codon (c.2270G>A; p.Trp757^*∗*^). This mutation, never described in the literature, permitted us to confirm the diagnosis of OPPG and it is the first case of OPPG among black Africans [9, 10, 12–14].

The mutation in the proband led to the production of a truncated protein containing 757 amino acids instead of 1615. In accord with previous studies [15, 16], the novel nonsense mutations reported here were equally located in the third *β*-propeller domain of the LRP5 protein.

**Figure 4:**
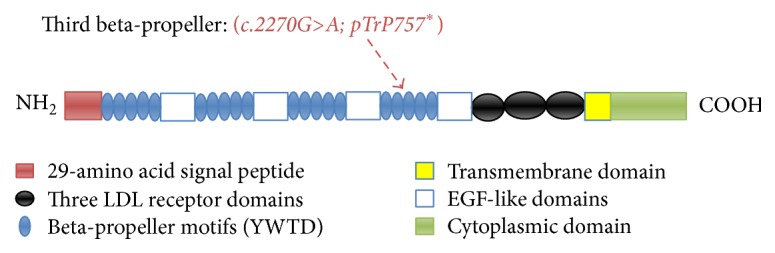
Schematic presentation of the protein structure and domain organization of LRP5. The novel LRP5 mutation is described here (shown in the schematic protein).

To simplify, the LRP5 protein contains a large extracellular domain (ECD), membrane-spanning domain, and an intracellular domain. The amino terminus of the extracellular domain (ECD) is followed by alternating beta-propeller motifs (YWTD), epidermal growth factor (EGF), and three LDL receptor domains [17]. The spanning domain is followed by a short intracellular domain (as shown in Figure 4). The YWTD is a binding domain that has a high affinity with* wnt* ligand [4, 5].

The p. Trp757^*∗*^ truncated protein containing the signal peptide, the first and second propeller (YWTD) domain, the first and second EGF-like domain, and a part of third *β* propeller domain (YWTD) (as shown in Figure 4), but lacking the transmembrane and cytoplasmic domains, which are crucial LRP5 protein regions [1], leading to degradation of truncated protein by the proteasome [6, 18].

For at least 14 different homozygous mutations and 16 compounds, heterozygous mutations have been described [1], with no phenotypic difference between homozygotes and heterozygotes in the literature [16, 19]. However, some heterozygous patients have been reported to have milder bone phenotype and normal eye phenotype [16]. The sever phenotype described in this report suggests that this novel mutation (c.2270G>A; pTrP757^*∗*^) is more pathogenic.

The pathogenic mechanism of OPPG is well understood: when wnt binds to Fz and LRP5, this allows beta-catenin stabilization, which interacts with gene transcription regulators. The above interactions lead to bone formation activation [16, 20]. Therefore, a mutation that prevents the connection between LRP5 and wnt will cause loss of function of the receptor, which results in OPPG syndrome [4, 19].

The function of LRP5 in eye development is complex [16]; however, many studies suggest that Lrp5 is also necessary for the normal regression of embryonic vasculature in the eye [11].

Several studies have shown the role of LRP5 gene in the acquisition of peak bone mass during growth [21, 22]. In addition to this, common polymorphisms of LRP5 have been associated with fracture risk and variations in BMD [23, 24]. Thus, thorough knowledge of OPPG can help us understand the physiology of bone tissue and therapeutic targets for osteoporosis.

In conclusion, we described the clinical and molecular features of a female Mauritanian child with OPPG due to a novel* nonsense* mutation in the LRP5. Our case expands the spectrum of LRP5 gene mutations in OPPG and highlights the important role of LRP5 in bone formation.
